# Impact of bitter tastant sub-qualities on retronasal coffee aroma perception

**DOI:** 10.1371/journal.pone.0223280

**Published:** 2019-10-03

**Authors:** Laurianne Paravisini, Ashley Soldavini, Julie Peterson, Christopher T. Simons, Devin G. Peterson

**Affiliations:** Department of Food Science and Technology, The Ohio State University, Columbus, OH, United States of America; The University of Tokyo, JAPAN

## Abstract

The impact of different bitter taste compounds on the retronasal perception of coffee aroma was investigated. A sorted napping experiment was carried out on thirteen compounds at iso-intense bitter concentrations. Differences in perceptual bitter sub-qualities among the compounds were reported by Multidimensional Scaling (MDS) and Multiple Factor Analysis (MFA) analyses. Seven exemplar compounds were further selected to investigate the impact of taste sub-qualities on cross-modal flavor interactions. In general, the different bitter compounds, when paired with a coffee aroma isolate, significantly modified the perception of the retronasal coffee aroma profile. Interestingly, the three bitter compounds endogenous to coffee had the most similar impact on the coffee aroma profile. Further sensory analysis of these sample sets indicated no significant effect of the bitter compounds on the orthonasal perception. Gas Chromatography/Mass Spectrometry (GC/MS) analysis of the volatile composition of the samples headspace also indicated negligible impact of the bitter compounds on aroma release. Altogether evidence of cross-modal interactions occurring at a higher cognitive level were demonstrated in a complex food sample, supporting the importance of multi-modal sensory integration on flavor perception.

## Introduction

Flavor is one of the most multimodal sensory experiences that humans perceive [1]. Multiple chemical stimuli are known to trigger our gustatory, olfactory and somatosensory receptor systems, generating inputs to the brain which are further processed into unique flavor percepts. The multisensory integration of flavor has been largely studied by sensory approaches [2–4] while others have used neuro-imaging techniques [5,6]. A recent “flavor network” model has been proposed [7] and gives the orbitofrontal cortex a central role in the integration of taste, aroma and chemesthetic signals although additional evidence suggests interactions might even occur in the periphery [8,9].

Multiple factors are known to impact cross-modal flavor interactions [10]. Taste and aroma interactions have been the most studied and known to result from physical, physiological, cognitive and/or psychological mechanisms [11]. In the context of food reformulation, odor enhancement by taste and taste enhancement by aroma have raised a lot of recent interest [12]. Sweetness, for example, has been notably reported as a main driver of overall flavor intensity in both model and real food systems [13–17]. Cognitive processes rather than physico-chemical interactions have been suggested as the main mechanisms for cross-modal interactions. For instance, evidence underscores the importance of congruency between stimuli for flavor interactions to be observed. Recent work demonstrated the significant effect of saltiness and umami stimuli on perceived intensities of chicken and soy sauce aroma; whereas the presence of bitterness had no effect [18]. The authors proposed that nutritive and/or beneficial substances, such as carbohydrates, in comparison to potentially toxic ones, such as bitter plant metabolites, can enhance a congruent odor. This was also previously proposed by Green and al. [13] suggesting that due to its non-nutritive character, a bitterness stimulus does not enhance the retronasal odor of foods.

Cross-modal interactions involving the impact of bitterness on a congruent aroma have not been studied. Additionally, very few studies have dealt with the impact of taste on the qualitative aspect of retronasal aroma despite its major role in the construction of the flavor object [19].

Understanding the influence of taste compounds on cross-modal flavor interactions can be further examined based on the taste sub-qualities. The perception of taste compounds ranges from uni- to multi-modal sensations. For example, sweeteners can exhibit additional sensory attributes other than sweetness [20]. Furthermore, differences in taste sub-qualities can be attributed to the temporal profile of the stimulus. Bitter perception (versus sweet or umami) is also unique due to the existence of multiple G-protein-coupled receptors and the broad tuning of the T2R receptors that are activated by hundreds of structurally different compounds [21]. Thus, humans have the ability to detect a wide range of bitter molecules from small ions to high molecular weight peptides. To this day, bitter activity cannot be predicted based on molecular structure. Previous work has identified clusters of compounds associated with different human detection thresholds; but the existence of different bitter qualities or the possibility of multiple bitter sub-qualities remains to be demonstrated [22].

The overall goal of this study was to investigate the mechanisms of taste-aroma interactions on flavor perception. Specifically, this work focused on characterizing how bitter tastants influence the retronasal perception of coffee aroma. To assess if a variety of bitter compounds differ in their bitter sub-quality perception, a sorted napping experiment was first employed. Coffee aroma was selected as a congruent stimulus and combined with different bitter tastants to evaluate the impact on retronasal aroma perception.

## Material and methods

### Chemical standards

Caffeine, (+)-catechin, (-)-epicatechin, naringin, sucrose octaacetate, L-tryptophan, L-phenylalanine, magnesium sulfate heptahydrate, sodium bicarbonate, quinine monohydrochloride dihydrate and methanol were purchased in food grade or primary reference standard quality from Sigma-Aldrich (St. Louis, MO). Urea was purchased from Spectrum Chemical (Gardena, CA). Sodium bicarbonate was purchased from Fisher Chemical (Fair Lawn, NJ). Cyclo(-leu-pro), cyclo(-pro-val), cyclo(-phe-pro) were synthetized by Bachem Americas (Torrance, CA) in high purity (>99%) and further underwent Solid Phase Extraction (SPE) to ensure high purity and removal of any residual solvent. Briefly, 0.6 g of material were dissolved in 200 mL of 90/10 water/methanol and loaded onto a 6g/35cc HLB Prime cartridge (Waters, Milford, MA). The cartridge was further washed with 60 mL of 95/5 water/methanol and elution was carried out with 60 mL of 95/5 water/methanol. Sample was freed from solvent and freeze-dried twice to ensure safety for consumption.

### High vacuum distillation of Arabica coffee brew: Solvent Assisted Flavor Evaporation (SAFE)

Roasted Arabica coffee beans (Ethiopia Harrar, Vienna roast) were purchased from a local coffee store (Columbus OH). Coffee brew was prepared from freshly ground coffee beans (40 g) and 200 ml of distilled water using a drip-coffee maker (Moccamaster KBT741, Technivorm, Italy). Volatile compounds were immediately extracted from fresh brew using Solvent Assisted Flavor Evaporation [23]. The internal SAFE apparatus was maintained at 40°C using a circulating water bath. The volatile compounds of the coffee brew were collected under high vacuum (approximately 6.10^−6^ Torr) over a two-hour period. The volatile isolate was kept at -40°C prior to analyses_._

### Personalized iso-intensity bitter solution

All human sensory trials were approved by the Ohio State University Institutional Review Board (#2017E0562 and #2017H0072).

**Fig 1:**
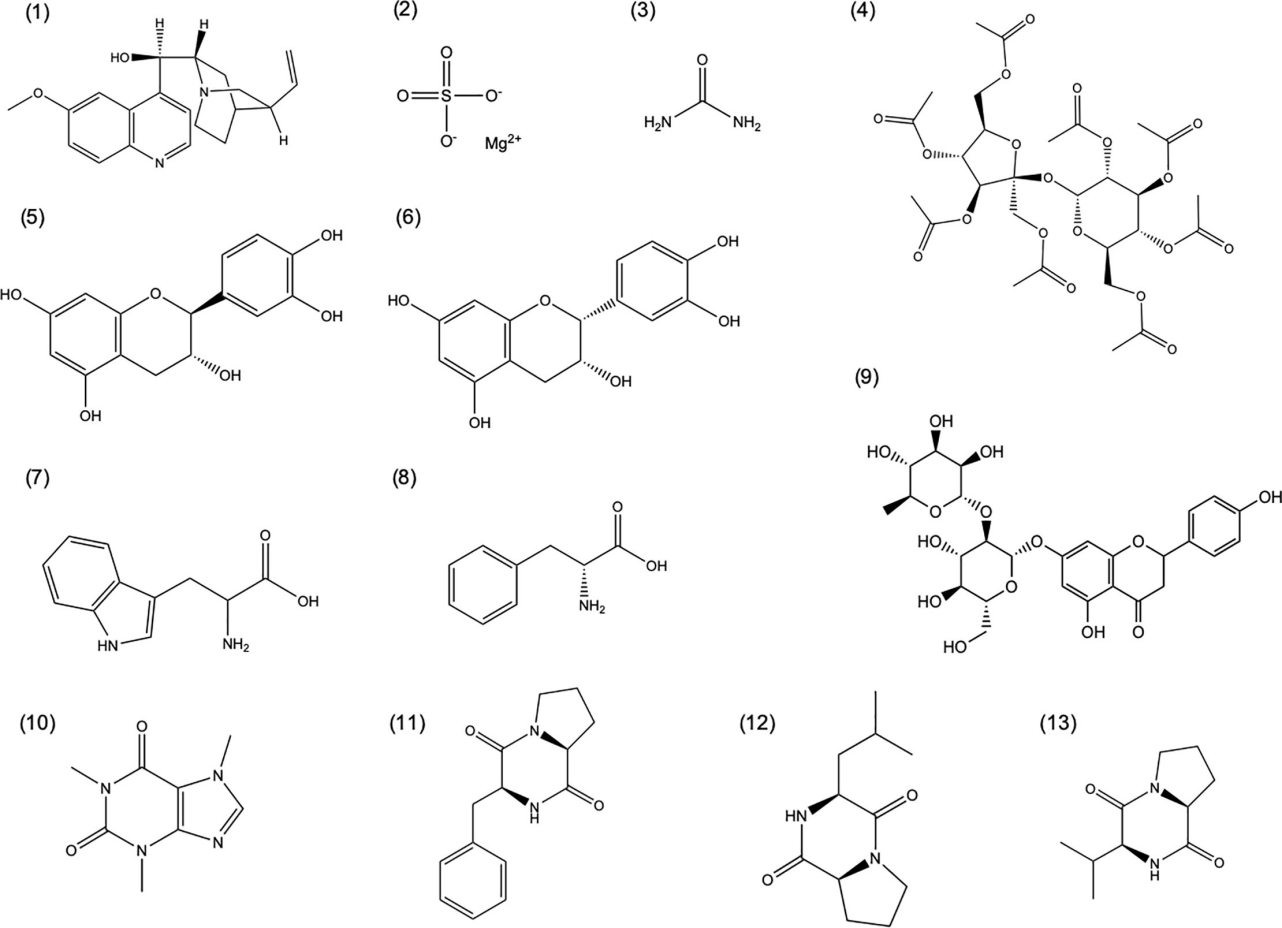
Selected bitter compounds (1) quinine, (2) magnesium sulfate, (3) urea, (4) sucrose octaacetate, (5) catechin, (6) epicatechin, (7) tryptophan, (8) phenylalanine, (9) naringin, (10) caffeine, (11) cyclo(-phe-pro), (12) cyclo(-leu-pro) and (13) cyclo(-pro-val).

Eleven panelists (7 females, 4 males; age range 24–35) were used in this experiment. For each panelist the concentration of each compound [Fig 1; caffeine, (+)-catechin, (-)-epicatechin, magnesium sulfate heptahydrate, naringin, L-phenylalanine, quinine hydrochloride dihydrate, sucrose octaacetate, L-tryptophan, urea, cyclo(phe-pro), cyclo(pro-val) and cyclo(leu-pro)] that elicited an equivalent bitter intensity was determined in two steps: reference determination and magnitude matching.

To determine each panelist’s reference sample concentration, individual participants tasted five caffeine solutions in increasing intensity (1–5 mM) and rated them using a general Labeled Magnitude Scale (gLMS) [24]. The caffeine solution rated between weak and moderate intensity became that panelist’s reference for the remainder of the protocol. In the second step, magnitude matching, panelists tasted 14 sets (13 compounds plus a blind control) of four solutions ranging from weak to moderate intensity. Panelists were instructed to taste each set of compounds following an increasing intensity order and to identify the solution that matched their specific weak/moderate caffeine reference sample. The individual selected sample concentrations for each panelist were used to develop the sorted napping sample sets. To minimize fatigue and carry-over effects, panelists rinsed with a salt solution (7% sodium chloride), followed by filtered water. Panelists also wore nose clips when tasting the compounds to ensure the activity focused solely on taste assessment.

### Sorted napping analysis of bitter compounds

The eleven panelists evaluated thirteen bitter compounds (caffeine, (+)-catechin, (-)-epicatechin, magnesium sulfate heptahydrate, naringin, L-phenylalanine, quinine hydrochloride dihydrate, sucrose octaacetate, L-tryptophan, and urea and cyclo(phe-pro), cyclo(pro-val) and cyclo(leu-pro)) at the determined iso-intensity concentration for each subject. Panelists were asked to taste each compound and sort them into groups based on sensory similarity. Using personalized stimulus sets in which bitterant concentrations were perceived as isointense ensured compound groupings were not based on intensity differences. Additionally, panelists were instructed to reflect the degree of sensory difference between samples (or groups) by adjusting the relative distance when placing them on a sheet of graph paper (25” X 30”, Post-it brand). Thus, compounds that tasted more similar were placed in closer proximity on the map. Data were first compiled in a similarity matrix, indicating the number of times each compound was placed in the same group as another compound. This matrix is used as input data for Multidimensional Scaling (MDS) analysis. Then, for each compound, the inter-group distances were calculated from the individual sorting maps. Specifically, the X and Y coordinates for each compound were determined by measuring the horizontal and vertical distance (in inches) from the left-hand corner of the graph paper to the sorted-group center. Panelists participated in a total of three tasting sessions: the initial individual titration of bitter compounds, a practice sorted napping session, and a sorted napping session for data collection. Multiple Factor Analysis (MFA) was carried out on the coordinates data using SensoMineR package in RStudio Version 1.1.456.

### Sensory descriptive analysis

Eight trained panelists (2 males, 6 females; age range 24–35) from the Flavor Research and Education Center at The Ohio State University were recruited to characterize the retronasal perception of coffee aroma. All panelists had previous experience with descriptive analysis and had participated in the previous titration and sorted napping studies. Term generation and panelist training occurred over 10 1-hour sessions. Attribute lexicon and references were chosen based on panelist consensus, using the World Coffee Research Lexicon as a guide [25]. The following attributes were evaluated: roasted, burnt, chocolate, caramelized, hazelnut, raisin, and brothy/meaty.

A unique set of samples for each panelist was prepared for evaluation consisting of the coffee aroma isolate and a bitter compound **[**caffeine, (+)-catechin, magnesium sulfate heptahydrate, quinine hydrochloride dihydrate, L-tryptophan, cyclo(pro-val) and cyclo(leu-pro)] at the individually determined iso-intense bitter concentration previously used in the sorting napping assessment. Samples consisted of 1 part of the SAFE coffee aroma isolate with 1 part of the bitter compound aqueous solution that was adjusted to pH 5.5 to mimic the pH of coffee using 0.1 M of sodium bicarbonate.

For evaluation, 20 ml of sample were presented in black 1-oz cups labeled with 3-digit codes. The seven samples were evaluated in duplicate in two sessions occurring over one day. Attribute intensities were rated using a 10-point line scale, anchored with 0 (not present) and 10 (strong). Data collection was performed using Compusense Cloud Software (Compusense Inc., Guelph, Ontario, Canada).

### Tetrad test for orthonasal evaluation of aroma

Fifteen trained panelists from the Flavor Research and Education Center at The Ohio State University participated in a series of seven tetrad tests to assess orthonasal differences between sample treatments. For each tetrad test, panelists were instructed to smell four samples from left to right and then sort the four samples into two groups of two based on similarity. Each tetrad test consisted of two control samples (coffee extract in water) and two test samples (coffee extract + bitterant). Presentation order was randomized across panelists. Samples (10 mL) were prepared as previously described, placed in 60 mL amber bottles and kept at room temperature until evaluation. Panelists participated in two sessions occurring over one day. Data were collected in duplicate using Compusense Cloud sensory analysis software and replicates were treated independently (n = 30, p<0.05).

### In-vitro aroma release analysis using Dynamic Headspace Gas Chromatography and Mass Spectrometry (DHS-GC/MS)

Volatile analyses were carried out on an Agilent 7890A GC (Agilent Technologies, Santa Clara, CA) equipped with a DB-Wax column (60 m × 0.250 mm × 0.25 μm, Agilent Technologies) and coupled with an Agilent 7010A Triple Quadrupole Mass Spectrometer (Agilent Technologies). The injector was equipped with a Thermal Desorption Unit (TDU, Gerstel, Mülheim an der Ruhr, Germany), PTV inlet (CIS 4, Gerstel), and a Multipurpose Sampler (MPS 2) with Dynamic Headspace (DHS) (Gerstel). Samples were prepared as previously described. Five hundred μL of sample were placed in 20 mL headspace vials and in incubation at 35°C for 5 min. Headspace was purged with nitrogen at a flow rate of 30 mL/min for a total volume of 200 mL. The trap (Tenax TA) was then dry purged at a rate of 60 mL/min for a total volume of 400 mL and transferred to a thermal desorption unit placed at 40°C. The volatile compounds were desorbed from the trap at 240°C for 2 min under a flow rate of 50 ml/min. Compounds were cryofocused on the CIS maintained at −75°C, then desorbed at 240°C for 3 min and then introduced onto the column using a 1:20 split ratio. The initial oven temperature was 50°C, ramped to 240°C at a rate of 5°C/min, and held for 5 min. Helium, as a carrier gas, was used at a constant flow rate of 1.2 mL/min. The mass scanning range was 29–400 *m/z* and mass spectra were obtained at 70 eV in the electron ionization (EI) mode. Compounds were positively identified using mass spectra, linear retention index (LRI) and injection of pure compound standards. LRI values were calculated with injection of n-alkane from C7-C30 in the same condition as the samples.

## Results and discussion

### Perceptual mapping of bitter compounds

A sorted napping experiment was conducted to evaluate if bitter taste compounds could be grouped based on sensorial similarities as well as to assess the relative difference between any observed groupings. Thirteen bitter compounds were selected (Fig 1) for evaluation, including: compounds 1–4: quinine (1), magnesium sulfate (2), urea (3) and sucrose octaacetate (4), known bitter compounds typically used as biological references in taste perception studies [26]; compounds 5–9: catechin (5), epicatechin (6), tryptophan (7), phenylalanine (8) and naringin (9) prevalent in food, plant materials and comprised of phenolic compounds and amino acids; and finally, compounds 10–13: caffeine (10) and three diketopiperazines (11–13) that have been previously identified in coffee and other natural products and processed foods [27].

For the sorted napping protocol, a titration procedure was used first to define individualized iso-intense bitter concentrations of compounds eliciting a weak to moderate intensity for each panelist (Table 1). This method accounted for the inter-individual variability in sensitivity for each compound evaluated. For example, review of the titration results indicated magnesium sulfate had the largest range of concentration perceived as iso-intense bitter, reporting a 24-fold difference between low and high values obtained from the panel (Table 1). In general, the results for the 12 remaining compounds showed that panelists ranged in sensitivities from 2 to 6-fold between panelists. Utilizing this titration step controlled for the noted individual variation in sensory responses among the panel that would be confounded by iso-intensity data calculated by averaging results from scaling methods.

**Table 1:** Individual titration (mM) procedure for intensity matching of bitter compounds.

Panelist	Concentration (mM)												
	Quinine	Magnesium sulfate	Urea	Sucrose octaacetate	Catechin	Epicatechin	Tryptophan	Phenylalanine	Naringin	Caffeine	Cyclo (-Phe-Pro)	Cyclo (-Leu-Pro)	Cyclo (-Pro-Val)
Panelist 1	0.021	85.2	140	0.008	4.1	4.1	7.1	24.5	0.15	1.0	4.1	1.9	5.1
Panelist 2	0.024	113.6	140	0.012	2.7	2.1	7.1	24.5	0.15	2.5	2.0	2.4	2.1
Panelist 3	0.014	42.6	210	0.006	2.4	2.7	10.6	16.4	0.15	3.5	2.0	2.4	5.1
Panelist 4	0.009	28.4	70	0.004	1.4	1.0	3.5	8.2	0.08	1.0	2.0	2.4	5.1
Panelist 5	0.012	42.6	70	0.004	1.4	1.4	3.5	16.4	0.08	1.5	2.0	1.2	3.6
Panelist 6	0.026	85.2	140	0.005	3.2	2.7	3.5	24.5	0.15	3.0	3.1	1.9	5.1
Panelist 7	0.014	56.8	280	0.006	2.7	2.1	10.6	20.5	0.11	2.5	2.0	2.4	5.1
Panelist 8	0.037	113.6	140	0.006	8.2	4.1	17.6	32.7	0.27	2.0	4.1	3.6	5.1
Panelist 9	0.019	4.7	70	0.012	2.1	1.4	7.1	16.4	0.38	3.0	2.0	2.4	5.1
Panelist 10	0.009	113.6	280	0.012	3.4	4.1	10.6	32.7	0.08	4.0	2.0	1.8	5.1
Panelist 11	0.014	113.6	140	0.008	1.43	4.8	10.6	24.5	0.13	4.0	4.1	3.6	5.1

The 13 compounds at the individual panelist iso-intense bitter concentrations were subjected to a sorted napping procedure that encompassed a sorting task based on overall similarity and a scaling task to further indicate their degree of difference from a defined origin [28]. The two-dimensional outcome of the napping experiments can be viewed as a limitation to represent complex perceptions such as flavor; however, multivariate statistical analyses are used post-hoc to help recover the full dimensionality of the stimuli [29]. Output data were collected in the form of a similarity matrix based on the frequency of grouping, as well as, spatial coordinates (*x*,*y*) unique to each sample/panelist combination. Both the frequency and coordinate data were further processed using Multidimensional Scaling (MDS) and Multiple Factor Analysis (MFA), respectively. Multidimensional Scaling (MDS) uses an iterative process to represent the measures of similarity/dissimilarity among pairs of samples as distances between points in a multidimensional space [30]. Multiple Factor Analysis (MFA), described as a weighted Principal Component Analysis (PCA), can be used to identify common spaces between individuals described by multiple variables such as aroma, taste and color [31]. For the MFA analysis, napping data comprised of the sample coordinates for each compound given by each panelist were considered as a group of variables [32].

**Fig 2:**
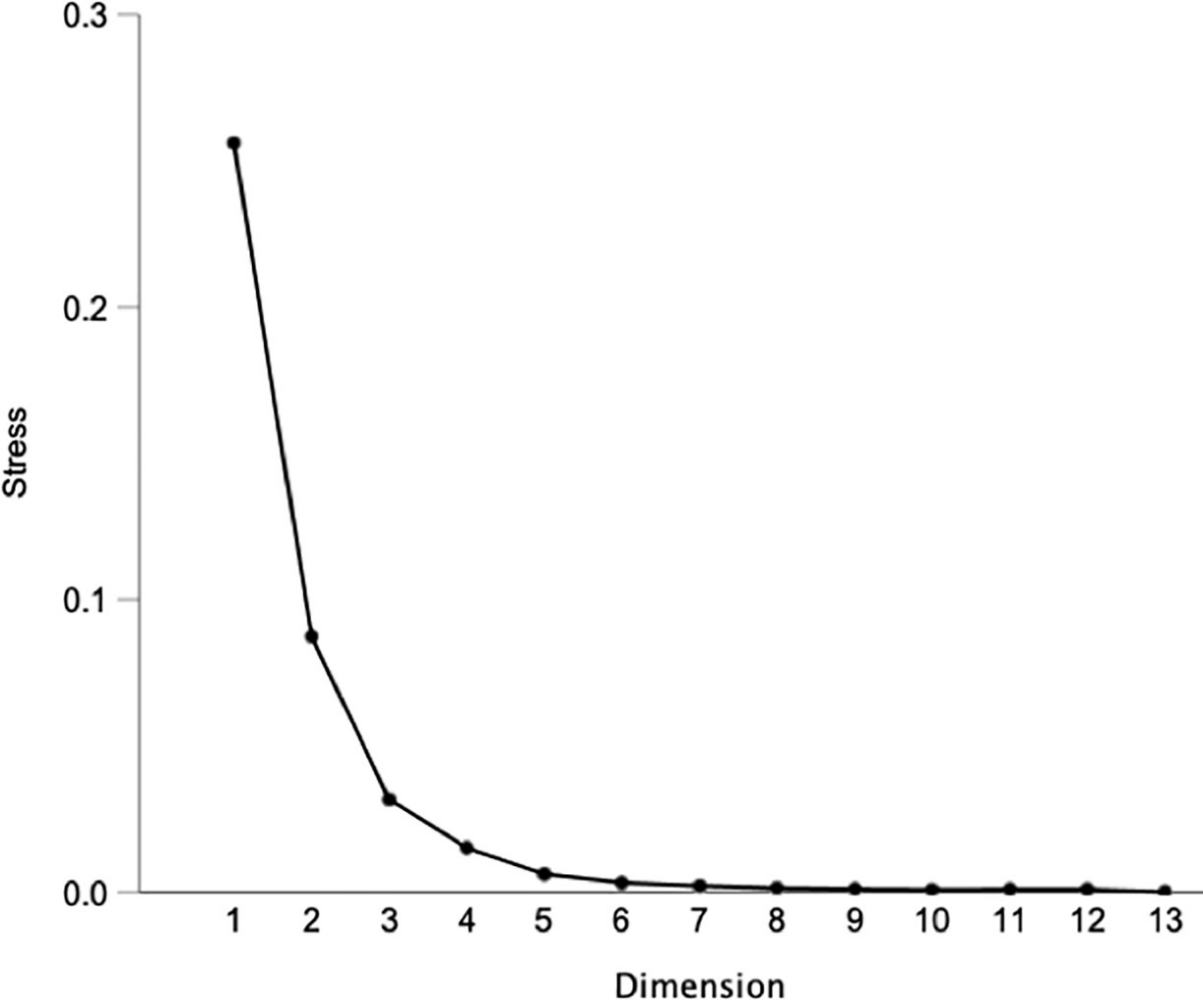
Scree plot for Multidimensional Scaling (MDS) of similarity matrix obtained from napping experiment.

The scree plot of the MDS given in Fig 2 represents the raw stress by dimensionality. In this study, three dimensions were retained as a meaningful reduction of stress was observed when they were added to the model. From this approach, the Kruskal’s type I stress value was computed and equaled to 0.18, indicating fairly good fit of the model [30].

**Fig 3:**
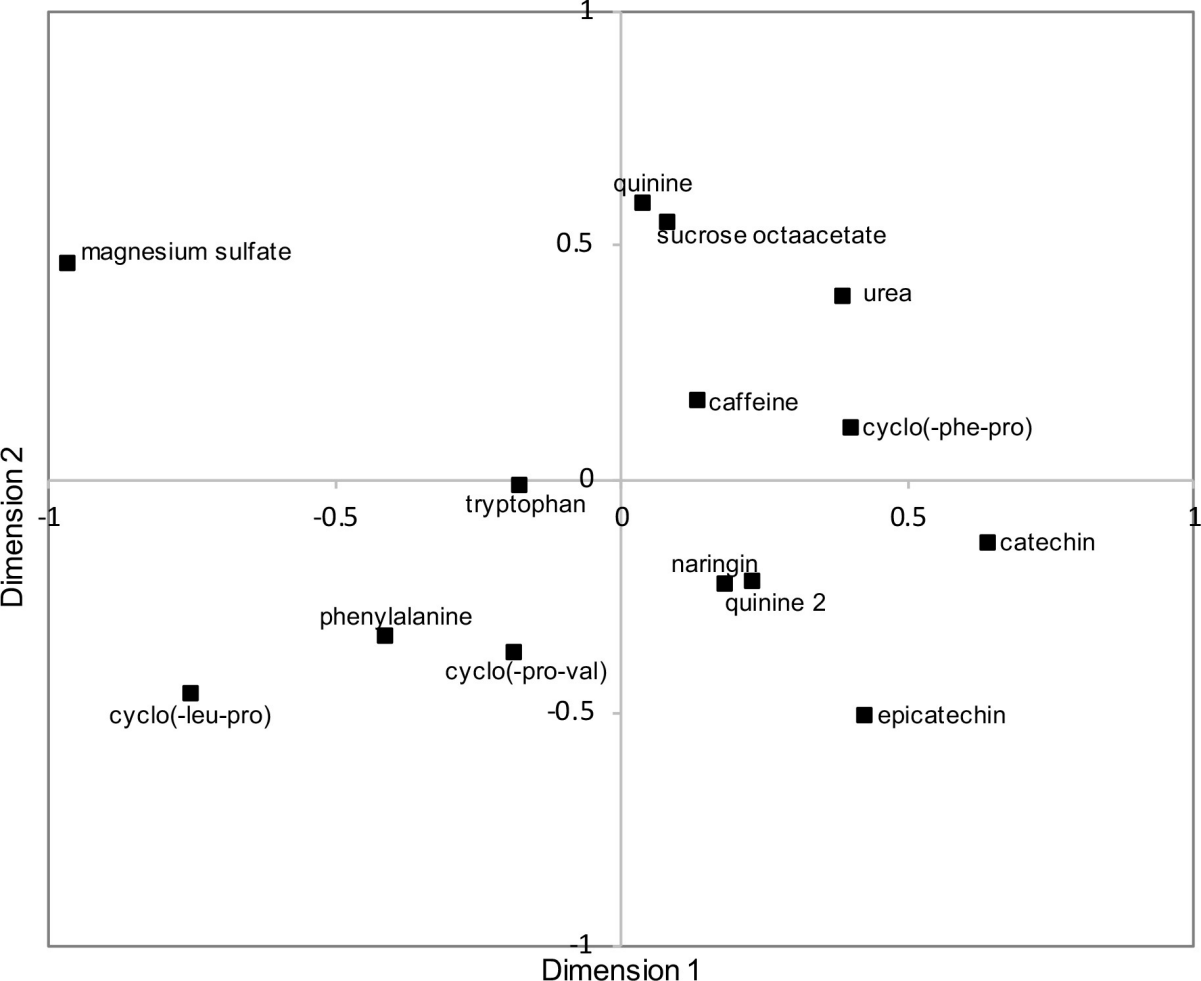
Perceptual map from Multidimensional Scaling (MDS) from similarity matrix obtained from napping experiments (Tucker’s coefficient of congruence = 0.984).

The output of the MDS is given on a perceptual map that represents the common space between samples (Fig 3). The distance between samples indicates their degree of similarity, i.e. the closer the samples are, the more similar they are perceived. Additionally, as the amount of variance explained on the first dimension (Dim 1) is larger than on the second dimension (Dim 2) (Fig 2), the differences observed on the horizontal direction are greater than differences in the vertical direction. Magnesium sulfate appeared as an outlier on the upper left suggesting greater differences from all the samples, and a unique taste sensory profile. Whether structural feature commonalities between compounds indicate sensory commonalities has not been elucidated [33], however the MDS plot revealed groupings based on chemical features and/or natural occurrence. Urea, sucrose octaacetate and quinine were located in the right top quadrant. These compounds are typically not found in food products. A second group composed of N-containing compounds was observed including tryptophan, phenylalanine, cyclo(-leu-pro) and cyclo(-pro-val) in the lower left quadrant. Naringin, catechin and epicatechin, common flavonoids, were equally spaced from each other and grouped in the lower right quadrant. Lastly, caffeine and cyclo(-phe-pro) constituted another group placed between the N-containing and the flavonoid group. Two quinine sample replications (quinine 1 and quinine 2), were given as blind replicates and results indicated close proximity on the first dimension, however on the second dimension one rep was close to sucrose octaacetate (top right quadrant) and the other rep was close to the flavonoids group (bottom right quadrant). The discrepancy arising from their separation on dimension 2 likely reflects a limitation either from the sensory task or from the statistical treatment that did not capture the complete similarities of the compound replicates. Nevertheless, the MDS plot showed groupings that suggested the existence of sub-qualities beyond bitterness allowing for the discrimination of the compounds.

**Fig 4:**
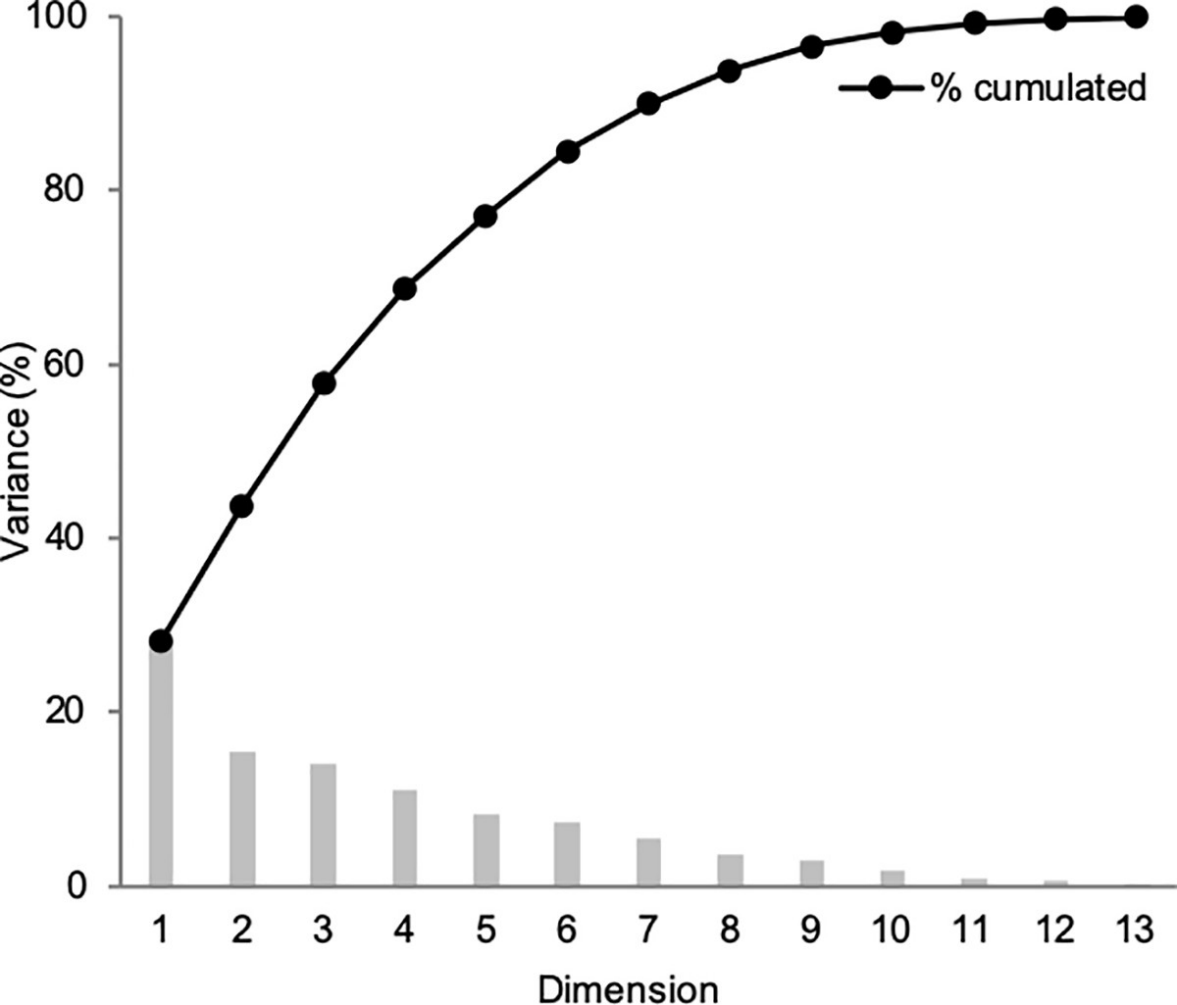
Scree plot for Multiple Factor Analysis (MFA) of sample coordinates obtained from napping experiment.

The MFA scree plot of the napping coordinate data is shown in Fig 4. The scree plot of the MFA differs from the MDS as it shows the variance explained by the multiple dimensions used to build the model. The first dimension (Dim 1) explained about 30% of the total data variance and dimensions 2 (Dim 2) and 3 (Dim 3) equally contributed, each explaining about 15% of the total variance. In order to represent most of the variance (> 80%), four dimensions were considered, which gave an indication of the complexity of the perceptual mapping space. This noted complexity further supports the MDS results suggesting the existence of sensory sub-qualities to the bitter compounds. In addition, panelist sensitivities to the different sub-qualities can vary; these interindividual differences are also likely contributing to the complexity of the data mapping.

**Fig 5:**
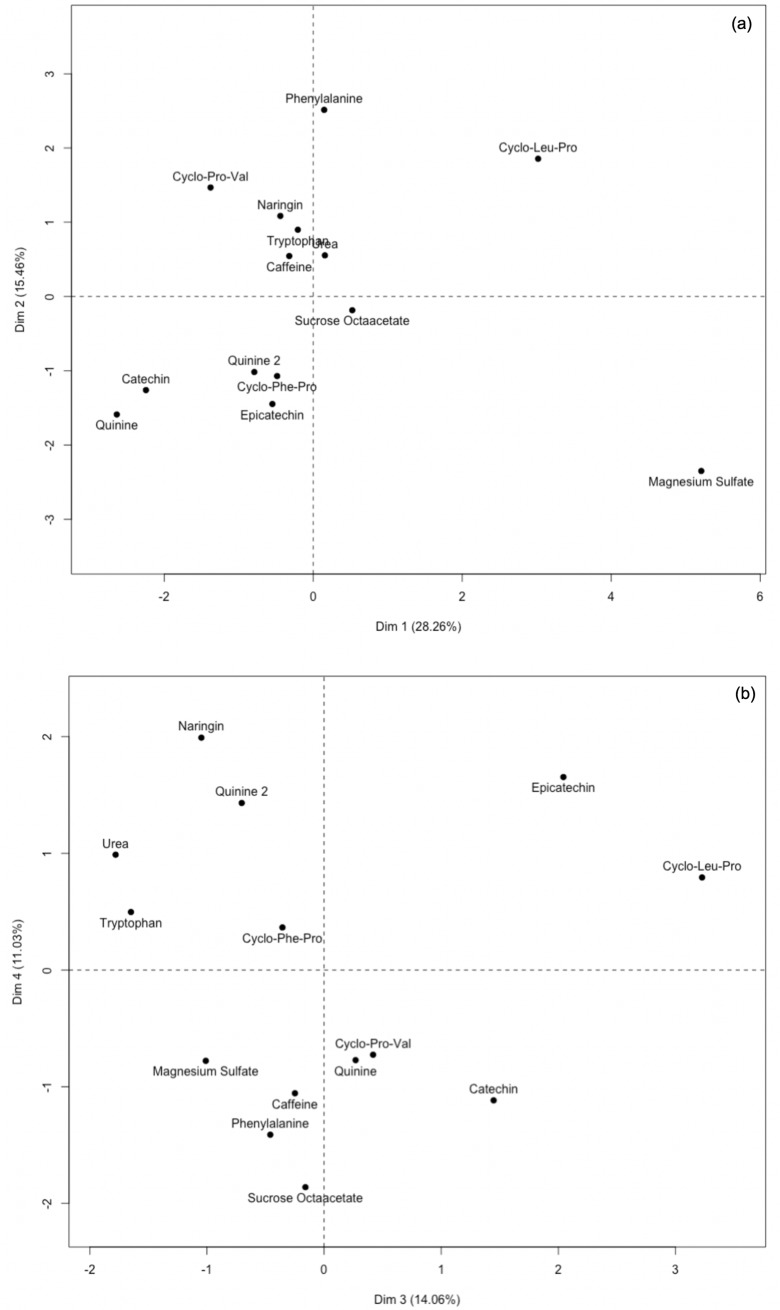
Multiple Factor Analysis (MFA) individual factor plots of (a) dimensions 1 and 2, (b) dimensions 3 and 4.

The MFA individual factor plots of the first four dimensions of the model are reported in Fig 5A and Fig 5B and show the spatial distribution of the compounds based on overall perception. First, the two samples of quinine, given as blind replicates, were closely represented on Dim 1 and 2 (Fig 5A) as well as Dim 3; but were differentiated along Dim 4 (Fig 5B). Considering the main dimensions of the MFA model as Dim 1, 2 and 3 (Fig 4), the results implied that the panelists were consistent in napping quinine and validated the sensory experiment as well as the statistical treatment to accurately represent the sensory experience. As depicted on Fig 4, Dim 4 contributed less to the model variance. In relation to the quinine results, this suggests that Dim 4 modeled noise or inconsistency in the data and might not be relevant for data interpretation.

On Dim 1, magnesium sulfate appeared as the most different (right), followed by cyclo(-leu-pro) (Fig 5A). This was highly indicative of a unique sensory profile associated with these compounds. This is in accordance with previous work in which magnesium sulfate appeared as an outlier possibly due to the presence of salty and sour sub-tastes that may have resulted in dumping effects and misuse of the bitter intensity scale [22]. The taste of metal salts have often been described as complex as that they do not generate a specific, well-defined sensation but rather one that is multifaceted that includes a bitter taste and additional attributes such as salty, metallic, astringent, sour and sweet, in order of decreasing intensity [34]. Similarly, other known bitter compounds, such as nicotine, have been recognized as both a bitter tastant and irritant [35,36], as well as capsaicin and menthol, known chemesthetic compounds that are also able to activate gustatory neurons that normally respond to bitter tasting substances [37]

The remaining 11 compounds appeared less differentiated on Dim 1 (left), but relevant clustering was identified in Dim 2 (Fig 5A). The first group included the quinine replicate samples, catechin, epicatechin and cyclo(-phe-pro) (bottom); caffeine, urea, tryptophan, naringin, cyclo(-pro-val) were grouped on the opposite side (top), and phenylalanine was the most differentiated at the extreme top (Fig 5A). On Dim 3, cyclo(-leu-pro), epicatechin and catechin (right) were separated from the remaining compounds that tend to overlap (left) (Fig 5B). Overall, the napping results (Fig 5A and Fig 5B) were aligned with the sorting results (Fig 3) and indicated significant grouping of the compounds according to sensory properties.

Prior work has indicated at least 25 receptors that respond to bitter compounds [21]. Whether these multiple pathways of transduction can elicit different bitter sub-qualities is not clear [38]. Prior psychophysical data indicate that bitter compounds may evoke sensations that are qualitatively different. Cross-adaptation studies have been used to indicate the degree of similarity between stimuli; the more complete the cross-adaptation, the more similar two compounds are in regard to their sensory quality [39]. Several studies have reported incomplete cross-adaptation between bitter taste compounds [39,40] indicating a lack of shared similarity in the coding mechanisms. Such results are also consistent with different compounds eliciting differing sensations. In accordance with these prior findings, the current work indicates that each of the 13 compounds did not elicit the same sensation, but rather exhibited a range of sensations or temporal profiles. For example, the three cyclo-peptide compounds (diketopiperazines) were represented at three different locations on the MFA plot (Fig 5) indicating different sensory profiles. Consistent with this finding, prior work reported the association of these cyclo-peptides with salty, metallic or astringent sub-tastes as well as differences in temporal attributes such as lingering [41]. The temporality of the perception, such as the rate of onset and lingering, can result in different overall sensory experiences. For example, at iso-intense bitter concentrations, caffeine perception greatly differs from quinine that has a faster build-up and a slower decay, resulting in a longer after taste [42]. Similarly, bitter catechins have been reported to exhibit a sweet after taste [43]which make them differ on both the quality and the temporal aspects.

### Impact of bitter compounds of retronasal coffee aroma perception

From the thirteen compounds analyzed for the taste perception analysis (Fig 3 and Fig 5), seven bitter compounds were selected that represented the range of sensory profiles observed and were used to further evaluate the impact of bitter qualities on retronasal aroma perception. Magnesium sulfate, cyclo(-leu-pro), cyclo(-pro-val), tryptophan, caffeine, catechin and quinine, at each individual panelist iso-intense bitter concentration, were combined with a coffee aroma distillate and the retronasal aroma perception was described using sensory descriptive analysis. Three of these compounds—cyclo(-leu-pro), cyclo(-pro-val), and caffeine have been found in coffee and, therefore more likely congruent stimuli for the coffee aroma.

The addition of bitter compounds to the coffee isolate had significant (p<0.001) and differential effects on the retronasal profile perceived by the trained panelists. Significant differences were found for all seven aroma attributes (Table 2) underscoring the significant role of the bitter compounds (at iso-intensity) on retronasal aroma perception. The replicate effect was not significant for any of the attributes indicating the consistency of the panel although the sample*panelist interactions were significant (Table 2). While this interaction can indicate misalignment, it is likely, in this case, to indicate that specific sub-qualities of the tastants uniquely modified individual panelist’s perception of the aroma or varied sensitivity among the panel toward the sub-taste qualities.

**Table 2:** Analysis of variance results from sensory descriptive analysis of retronasal coffee aroma and mean intensities (± Standard Error) (n = 8).

Sample	Aroma descriptors						
	brothy/meaty	burnt	caramelized	chocolate	hazelnut	raisin	roasted
Caffeine	1.2 ± 0.2^ab^	5.8 ± 1.2^cd^	2.1 ± 0.6^a^	5.1 ± 1.2^c^	2.6 ± 0.4^a^	2.8 ± 0.7^abc^	5.0 ± 0.6^a^
Catechin	0.8 ± 0.2^a^	4.7 ± 1.3^bc^	2.1 ± 0.4^a^	2.9 ± 0.5^a^	2.8 ± 0.6^a^	2.5 ± 0.6^abc^	3.1 ± 0.6^b^
Cyclo(-leu-pro)	1.8 ± 0.5^b^	6.5 ± 0.9^d^	2.6 ± 1.2^a^	4.6 ± 0.7^bc^	2.5 ± 0.9^a^	2.3 ± 0.7^a^	4.4 ± 0.5^a^
Cyclo(-pro-val)	1.7 ± 0.3^ab^	6.1 ± 1.1^cd^	3.0 ± 0.8^ab^	5.2 ± 0.9^c^	3.1 ± 0.6^ab^	2.3 ± 0.4^ab^	4.4 ± 0.7^a^
Magnesium sulfate	2.0 ± 0.6^b^	2.7 ± 0.7^a^	5.0 ± 0.7^c^	3.1 ± 0.8^ab^	4.0 ± 0.9^c^	3.9 ± 0.7^d^	4.3 ± 0.7^a^
Quinine	1.5 ± 0.6^ab^	4.1 ± 0.7^ab^	3.9 ± 0.5^a^	5.1 ± 0.8^c^	3.2 ± 0.7^bc^	1.5 ± 0.8^bcd^	4.1 ± 1.1^a^
Tryptophan	2.1 ± 0.6^b^	3.8 ± 0.6^ab^	3.6 ± 1.0^b^	4.1 ± 1.0^abc^	4.1 ± 0.7^c^	3.5 ± 0.8^cd^	5.1 ± 0.8^a^
Effect	*p-value*						
*Sample*	*0*.*0015*	*<* .*0001*	*<* .*0001*	*<* .*0001*	*<* .*0001*	*<* .*0001*	*<* .*0001*
*Panelist*	*<* .*0001*	*<* .*0001*	*<* .*0001*	*<* .*0001*	*<* .*0001*	*<* .*0001*	*<* .*0001*
*Rep*	*0*.*32*	*0*.*63*	*0*.*56*	*0*.*69*	*0*.*31*	*0*.*57*	*0*.*24*
Sample*Panelist	*0*.*0044*	*0*.*0003*	*<* .*0001*	*0*.*0003*	*<* .*0001*	*<* .*0001*	*<* .*0001*

*Within each column, different letters indicate significant differences according to post-hoc Tukey HSD (α = 0.05)

**Fig 6:**
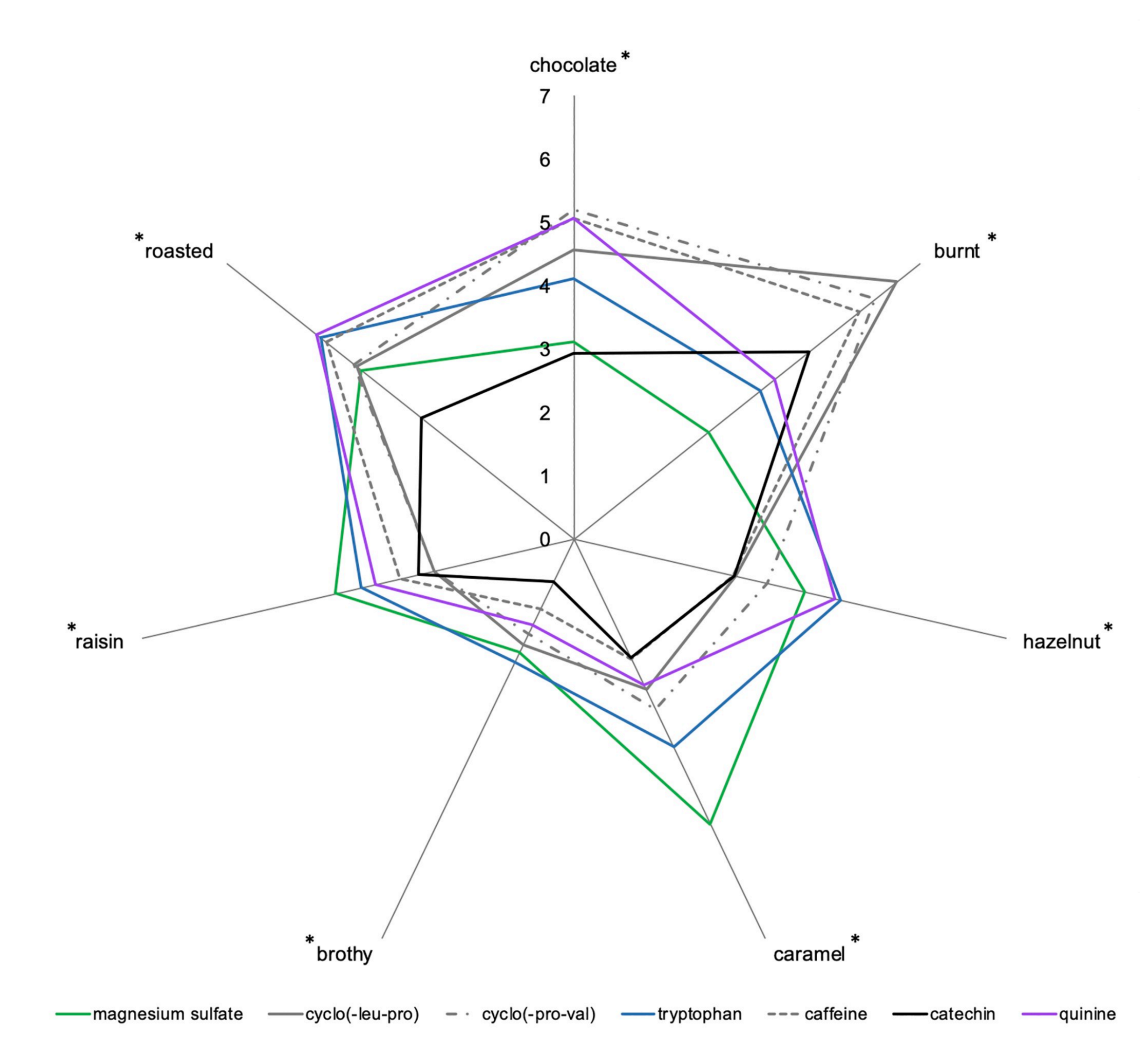
Retronasal descriptive analysis of coffee aroma attribute intensities in seven bitter compounds. * indicates significant impact of bitter compounds on aroma attribute intensity (α = 0.05).

The radar plot of the attribute intensities of the seven bitter compounds is shown in Fig 6. Review of the descriptive analysis indicated significant impact of bitter compounds on retronasal aroma perception. Interestingly, the three endogenous coffee bitter compounds, and thought to be most congruent with the coffee aroma profile, elicited the most similar profile (see radar plots of cyclo(-leu-pro), cyclo(-pro-val), and caffeine in Fig 6). This underscores the importance of congruency in cross-modal taste-aroma interactions that have been noted previously [11,13,17,18]. On the other hand, with the addition of other bitter compounds, a large range of perceived intensities was observed. This was particularly true for the attributes burnt and caramelized that exhibited up to a four point (out of 10) difference between samples. Based on Tukey HSD post-hoc multiple comparison tests (α = 0.05), up to four groupings (a-d) were noted among the seven compounds. The burnt aroma was most prominent in cyclo(-leu-pro) (group a) rated at 6.5 and conversely, magnesium sulfate sample exhibited the lowest intensity at 2.7 (group d). The five other samples were rated intermediately with intensities ranging from 3.8 to 6. The caramel attribute exhibited the second greatest change with the highest intensity for the magnesium sulfate sample rated at 5 (group a). The catechin, caffeine, quinine and cyclo(-leu-pro) samples were equally rated the lowest with an intensity of about 2.3 (group c). The attributes roasted, raisin, brothy, hazelnut and chocolate showed the lowest extent of changes, however significant differences in intensities were observed between samples.

Comparison of the MFA results (Fig 5) with the noted changes in the aroma profiles (Fig 6) indicated agreement between the results. For example, magnesium sulfate, quinine and cyclo(-leu-pro), which elicited the most different taste profiles according to Dim 1 and 2 of the MFA (Fig 5A), were also found to differ the most in their impact on the perceived intensity of the burnt aroma attribute. Similar results were observed with the hazelnut attribute which showed the lowest intensity for cyclo(-leu-pro), whereas magnesium sulfate and quinine were rated similarly high (Fig 6). Further review of Dim 3 (Fig 5B) indicated cyclo(-leu-pro) was clearly differentiated, whereas magnesium sulfate and quinine appeared more similar. Hence, alignment between noted changes in the taste qualities (Fig 5) and the aroma ratings (Fig 6) for these particular combinations of aroma/tastants was reported. Additionally, similar to the mapping experiment results, the magnesium sulfate sample that showed the most unique flavor profile when tasted without aroma (Fig 5A), also showed the most unique aroma profile (Fig 6). The retronasal aroma for the magnesium sulfate sample was described with the lowest intensities of chocolate and burnt aromas and the highest intensities of the remaining attributes. Thus, the unique taste sub-qualities and/or somatosensory properties of magnesium sulfate could explain the largest observed change in the coffee aroma profile perceived. This could be explained by the sub-qualities of magnesium sulfate decreasing the congruency with chocolate and burnt aroma attributes, resulting in lower intensity ratings. However, descriptive profiles of the subqualities of the tastants are needed to support this finding. In general, the more extreme (lowest or highest) intensity values for the aroma attributes were consistently given following addition of either magnesium sulfate, catechin, or cyclo(-leu-pro) to the coffee isolate.

In general, the sensory descriptive analysis results indicated the significant role of the different bitter compounds (sub-qualities) on the retronasal perception of coffee aroma, supporting the importance of cross modal interactions on flavor perception. It is possible, although not anticipated, the altered retronasal aroma perceptions are the consequence of different release of volatile compounds in the headspace that result from different chemical-physical interactions with each bitter compound. To test for possible molecular interactions, the bitter compounds at their highest concentration, as determined in the titration procedure (Table 1), were recombined individually with the coffee aroma and the volatile aroma release was further analytically monitored using Gas Chromatography/Mass Spectrometry (GC/MS) (Table 3). Twenty volatile compounds of the coffee extract that ranged in volatility and chemical class were monitored. Overall, no significant differences in aroma release into the headspace vials were observed for 18 compounds out of 20 (α = 0.05). A significant effect of bitter compounds on release was only observed for dihydro-2-methyl-3(*2H*)-furanone and pyridine with 18 and 25% differences in the headspace between the extreme samples, respectively. Hence 95% of the coffee volatile compounds release profile were not impacted by the addition of bitter compounds. This indicates that addition of the bitter compounds, at these levels, is unlikely to result in physico-chemical interactions able to modify the aroma perception. Furthermore, the two compounds [dihydro-2-methyl-3(*2H*)-furanone and pyridine] were only significantly different in 1 or 2 samples (Table 3), with the majority of the samples being not significantly different. Among the samples that reported no significant differences in the aroma headspace concentration [catechin, tryptophan, caffeine, cyclo(-leu-pro), and cyclo(-pro-val], significant changes in the aroma profile were still observed (Table 2).

**Table 3:** Peak areas (GC/MS) of coffee volatile compounds in bitter matrices (caffeine, catechin, cyclo(-leu-pro), cyclo(-pro-val), magnesium sulfate, quinine and tryptophan).

DB_Wax retention index calculated	DB_Wax retention index NIST 2.3	Volatile Comppunds	Peak Area							Bitter compound effect*
			quinine 0.037mM	Mg sulfate 113.6mM	catechin 8.2mM	tryptophan 17.6mM	caffeine 4mM	cyclo (-leu-pro) 3.6mM	cyclo (-pro-val) 4.1mM	
1202	1185	pyridine	5.4E+08^c^	5.4E+08^bc^	7.3E+08^abc^	7.1E+08^ab^	6.3E+08^a^	7.1E+08^ab^	7.2E+08^a^	*
1278	1268	dihydro-2-methyl-3(*2H*)-furanone	1.9E+07^b^	2.1E+07^ab^	3.1E+07^ab^	2.6E+07^ab^	2.3E+07^a^	2.9E+07^ab^	3.0E+07^ab^	*
1285	1282	2-methylpyrazine	1.1E+08	1.3E+08	1.7E+08	1.4E+08	1.3E+08	1.5E+08	1.5E+08	ns
1327	1321	1-hydroxy-2-propanone	2.3E+07	2.1E+07	2.1E+07	2.4E+07	1.5E+07	2.1E+07	2.1E+07	ns
1343	1333	2,5-dimethylpyrazine	8.2E+06	8.2E+06	5.5E+06	7.2E+06	6.8E+06	7.0E+06	5.9E+06	ns
1347	1339	2,6-dimethylpyrazine	6.1E+06	6.3E+06	6.4E+06	7.0E+06	6.9E+06	7.1E+06	6.6E+06	ns
1347	1343	ethylpyrazine	2.5E+07	2.8E+07	2.9E+07	2.6E+07	2.4E+07	2.7E+07	2.8E+07	ns
1399	1395	2-ethyl-6-methylpyrazine	1.8E+07	1.9E+07	2.6E+07	2.4E+07	2.1E+07	2.1E+07	2.2E+07	ns
1423	1422	2-ethyl-3-methylpyrazine	7.3E+06	7.9E+06	8.5E+06	7.6E+06	7.0E+06	8.1E+06	8.6E+06	ns
1459	1444	2,6-diethylpyrazine	7.8E+06	9.7E+06	8.4E+06	9.0E+06	7.2E+06	8.0E+06	9.7E+06	ns
1483	1480	furfural	1.1E+08	1.2E+08	1.1E+08	9.8E+07	1.3E+08	1.6E+08	1.6E+08	ns
1519	1510	2-acetylfuran	5.6E+07	6.0E+07	7.7E+07	6.4E+07	6.0E+07	7.0E+07	7.3E+07	ns
1544	1542	furfuryl acetate	7.8E+07	8.4E+07	9.9E+07	9.7E+07	8.2E+07	9.8E+07	1.0E+08	ns
1593	1597	5-methylfurfural	1.1E+08	1.2E+08	1.3E+08	1.0E+08	1.2E+08	1.3E+08	1.4E+08	ns
1640	1626	N-methyl-2-formylpyrrole	2.7E+07	2.8E+07	3.3E+07	3.1E+07	2.6E+07	3.1E+07	3.2E+07	ns
1669	1660	furfuryl alcohol	1.3E+08	1.4E+08	1.6E+08	1.4E+08	1.4E+08	1.4E+08	1.4E+08	ns
1674	1660	2-acetyl-1-methylpyrrole	8.9E+06	9.2E+06	1.0E+07	9.5E+06	8.4E+06	9.5E+06	9.9E+06	ns
1839	1824	furfurylpyrrole	8.4E+06	8.5E+06	7.9E+06	9.6E+06	7.6E+06	8.8E+06	9.3E+06	ns
1876	1860	guaiacol	1.1E+07	1.2E+07	1.2E+07	1.1E+07	1.1E+07	1.2E+07	1.2E+07	ns
2017	2008	phenol	9.3E+06	1.0E+07	1.1E+07	1.0E+07	1.1E+07	1.0E+07	9.5E+06	ns

*indicates significant effect of bitter compound (α = 0.05).

Different letters indicate significant differences according to Tukey posthoc.

Further sensory orthonasal analysis was conducted to investigate the impact of any physical-chemical interactions for each bitter compound on aroma perception. Samples of coffee aroma with and without each of the seven bitter compounds were evaluated using a tetrad test. The results showed that panelists were unable to differentiate the coffee isolates with or without added bitter compounds (Table 4) indicating that the orthonasal coffee aroma perception was not impacted by the addition of bitter compounds. These results support the prior analytical results and indicated that physico-chemical interactions did not alter the aroma headspace composition and thus were not the primary mechanisms responsible for the observed changes in retronasal aroma perception (Fig 6). Based on the spatial isolation of taste and aroma receptors in the oral and nasal cavities, respectively, peripheral interactions at the receptor level would not occur, and suggests a mechanism whereby these modulatory effects occur at the cognitive level.

**Table 4:** Results of tetrad test for orthonasal evaluation of coffee aroma in seven bitter compounds and compared to water.

Bitter compounds	Number of correct answers (n = 30)	p-value*
Catechin	14	0.09
Quinine	14	0.09
Caffeine	9	0.71
Magnesium sulfate	13	0.17
Cyclo(-leu-pro)	14	0.09
Cyclo(-pro-val)	14	0.09
Tryptophan	13	0.17

* corresponding probability for numbers of correct answers for 30 trials and 1/3 chance

In summary, the findings of this study support the integration of taste and aroma stimuli that result in unique flavor percepts that have been described previously [7]. In this work, altering the bitter stimulus was demonstrated to modulate the aroma inputs resulting in a modified flavor percept. Overall, these findings demonstrate the importance of multi-integration of flavor inputs for perception.
